# Retinitis pigmentosa associated with blepharophimosis, blue dot cataract and primary inferior oblique overaction: A new syndrome complex or consummate myotonic dystrophy?

**DOI:** 10.4103/0301-4738.53068

**Published:** 2009

**Authors:** Pramod Kumar Pandey, Pankaj Vats, Pooja Jain, Ashish Amar, Yuvika Bansal

**Affiliations:** Guru Nanak Eye Centre, Maulana Azad Medical College, University of Delhi, New Delhi - 110 002, India

Dear Editor,

We thank the authors for their equivocations in reply to our letter.[1] The reply fails to address the inherent contradictions cloaking the report, be it clinical findings, diagnostic oversights or syndromic prophecy, implicitly paraphrasing blepharophimosis (BP), euryblepharon, V pattern strabismus, anatomy and embryology of the lids and extra-ocular muscles (EOMs) as well as association between BP, epicanthus inversus and ptosis syndrome (BEPS) and strabismus.

In BP, the horizontal lid apertures range between 18 to 22mm, apertures from 25 to 30mm are taken as normal.[2] Apertures of 26 and 28mm here, do not constitute BP, BP and euryblepharon, BP and euryblepharon are contradictory and cannot coexist as reported, similarly exophoria in primary position and divergence in upgaze implies a T rather V pattern.[3] Versions here betray an unmistakable asymmetrical limitation of both upgaze and downgaze, simulating simultaneous overaction of both inferior and superior oblique muscles and underaction of vertical recti due to myotonia and/or myopathy.[3] BP, BEPS are thus out of bounds, so are the hallowed, prophesied syndromic affiliations with syndromic retinitis pigmentosa.

The constellation of findings[3] instead, constitute seminel manifestations of ocular involvement in myotonic dystrophy (MD)-1. Spectrum of presentation in MD, mirrored in this case may include ptosis, asymmetric motility limitations; blue, blue-green, iridescent dust/dots, snowballs, cortical spokes, subcapsular plaques in the lens; attenuated retinal arterioles, peripheral pigmentary degenerations, diminished/extinguished photopic/ scotopic responses on electroretinograph and peripheral constriction of visual fields.[4] Optic atrophy has been described on many occasions.[5] A pupil refractory to dilatation[3] bears mute testimony to the underlying disorder. Enophthalmos, seborrheic blepharitis, neovascular tufts on the iris, short depigmented ciliary processes, eso/exotropia, convergence insufficiency, epiphora, dry eye, hypotony and pigment streaks at fovea are also reported.[4] MD-1 is an autosomal dominant disorder resulting from an expansion of unstable cytosine, guanine, thymidine (CTG) repeat in the 3′- untranslated region of a protein kinase gene (DMPK) on chromosome 19q13.3.[5] MD-2 results from abnormalities on chromosome 3. Normals have 5 to 30 CTG repeats; patients with MD-1 have 50 to 3000 repeats. More repeats imply more severe disease. Variability in presentation; reduced smooth pursuit gain and reduced saccadic peak velocity are hallmarks in MD, a multi-system disorder involving muscular, ocular, endocrine, cardiac and cognitive impairment.

Mesodermal dysgenesis and embryogenesis of EOMs have nothing proximate between them,[1] other than primordial mesodermal underpinnings, how authors drew a parallel between the two, imputing culpability on us is intriguing.

BEPS and strabismus are related, contrary to authors’ assertions.[1] Davson *et al*.,[6] document strabismus in 20% of cases of BEPS.

Notwithstanding authors assertions,[1] other than epidermis, epidermal appendages including eye-lashes, glands and conjunctival epithelium, the rest of eyelids including dermis, orbicularis oculi, tarsal plates, levator palpebrae superioris, substantia propria of the conjunctiva and blood vessels are strictly mesodermal in origin.

The authors need to be congratulated for appalling ingenuity in conflating a new syndrome[3] in the genre of syndromic retinitis pigmentosa from a century old MD[4] Mis-diagnosing MD and eschewing interdisciplinary approach can be parlous, as arrythmias can lead to sudden death, raising medico-legal and ethical issues for the ophthalmologist.
